# Risk Factors for Atorvastatin as a Monotherapy for Chronic Subdural Hematoma: A Retrospective Multifactor Analysis

**DOI:** 10.3389/fnagi.2021.726592

**Published:** 2021-09-01

**Authors:** Xinjie Zhang, Dong Wang, Ye Tian, Huijie Wei, Xuanhui Liu, Tangtang Xiang, Yibing Fan, Chuang Gao, Jinhao Huang, Zhuang Sha, Wei Quan, Jianning Zhang, Rongcai Jiang

**Affiliations:** ^1^Department of Neurosurgery, Tianjin Medical University General Hospital, Tianjin, China; ^2^Tianjin Neurological Institute, Key Laboratory of Post-Neuroinjury Neuro-Repair and Regeneration in Central Nervous System, Tianjin Medical University General Hospital, Ministry of Education, Tianjin, China

**Keywords:** chronic subdural hematoma, atorvastatin, conservative therapy, head trauma, aging, risk factor analysis

## Abstract

Chronic subdural hematoma (CSDH) is a common form of intracranial hemorrhage in the aging population. We aimed to investigate the predictive factors for atorvastatin efficacy as a monotherapy for moderate CSDH. We retrospectively reviewed the medical records of patients who were diagnosed with moderate CSDH and received atorvastatin monotherapy between February 5, 2014, and November 7, 2015, in multiple neurosurgical departments. Univariate, multivariate and receiver operating characteristic curve analyses were performed to identify the potential significant factors indicative of the good therapeutic efficacy or poor therapeutic efficacy of atorvastatin for mild CSDH, such as age, sex, history of injury, Markwalder grading scale–Glasgow Coma Scale (MGS-GCS), Activities of Daily Life-the Barthel Index scale (ADL-BI), American Society of Anesthesiologists Physical Status classification system (ASA-PS), blood cell counts, serum levels and computed tomography findings. A total of 89 patients (75 men and 14 women) aged 24–88 years (mean age 61.95 ± 15.30 years) were followed-up for 24 weeks. Computed tomography findings at admission showed mixed-density hematoma in 22 patients, isodense hematoma in 13 patients, high-density hematoma in 26 patients, and low-density hematoma in 28 patients. In total, 3, 80, and 6 patients had MGS-GCS grades of 0, 1, and 2, respectively. The efficacy rate at 6 months was 87.6% (78/89). Eleven patients were switched to surgery due to a worsened neurological condition, of whom 8, 1, 1, and 1 had high-density, low-density, isodense and mixed-density hematomas, respectively. These patients were switched to surgery over a range of 2–27 days, with a median interval of 12 days after the medication treatment. Univariate and multivariate analyses, confirmed by ROC curves, revealed that high-density hematoma, basal cistern compression, and hematoma volume to be independent risk factors for the efficacy of atorvastatin monotherapy in patients with moderate CSDH. Atorvastatin is an effective monotherapy for the treatment of mild CSDH. High-density hematoma, basal cistern compression, and hematoma volume are independent predictors of the efficacy of atorvastatin as a non-surgical treatment. The results suggested that ADL-BI was more sensitive than the MGS-GCS and ASA-PS for determining patient outcomes in our moderate CSDH cohort.

## Introduction

Chronic subdural hematoma (CSDH) is increasingly common because of the aging population (Liu et al., 2014). The total estimated incidence is 13.5 per 100,000 persons per year in the general population, and for those above the age of 65 years, it can be up to 58.1 per 100,000 persons per year (Kudo et al., 1992). Burr-hole drainage with or without rinsing is the first choice of treatment. However, the incidence of recurrent CSDH may reach 25%, regardless of the type of surgery. Furthermore, most CSDHs occur in older patients, who carry a high risk of perioperative infection, pneumonia and high-surface-tension pulmonary edema (Santarius and Hutchinson, 2004; Muzii et al., 2005). An overall mortality rate of 38.4% is reported for patients 90 years or older, independent of the treatment approach (Lee et al., 2016). Thus, safe and effective non-surgical treatments are needed. Atorvastatin, a 3-hydroxy-3-methylglutaryl (HMG)-coenzyme A reductase inhibitor, which is a type of lipid-lowering medication. It has been found to promote neovascularization with vascular maturation at the neomembrane of CSDH and inhibition of inflammation (Verschuren et al., 2005; Araújo et al., 2010; Wang et al., 2016). The formation of mature vessels at the neomembrane reduces vascular leakage and hence preventing hematoma progression. At the same time, the formation of mature vessels was observed to be associated with subdural hematoma absorption (Wang et al., 2010; Li et al., 2014). Furthermore, our previous randomized placebo-controlled trial, on atorvastatin (ATO), was shown to be safe and effective in reducing CSDH and improving the neurologic dysfunction of adult patients (Jiang, 2018). Nonetheless, to our knowledge, neither original articles nor reviews focused on the risk factors for atorvastatin as a monotherapy in the treatment of CSDH have been published thus far. Previous studies have already reported the use of corticosteroids, angiotensin converting enzyme inhibitors, and tranexamic acid as adjuncts to surgery. However, the majority of these studies were single-center retrospective studies with interactions between many variables and confounding factors, especially surgical elements (Berghauser Pont et al., 2012; Ivamoto et al., 2016; Fujitani et al., 2017). We therefore attempted to investigate the predictive factors for atorvastatin monotherapy for mild CSDH and to provide more insights into the use of atorvastatin therapy for CSDH.

## Materials and Methods

### Patients

We retrospectively reviewed the consecutive medical records of patients who received a diagnosis of chronic subdural hematoma between February 2014 and November 2015 across 25 neurosurgical departments. The study was approved by the ethics committees of the participating hospitals. The MGS and GCS scores and ADL-BI (Activities of Daily Life-the Barthel Index) were measured at admission (Kwok et al., 2011). The ADL-BI evaluates the ability of a patient to dine, bathe, groom (face washing, tooth brushing, shaving and combing), dress (tying shoes and fastening buttons), defecate, urinate (self-cleaning, adjusting clothing, and washing up), go to bed and move a chair, walk on a flat floor and go up and down a flight of stairs. Complications and adverse events were recorded.

### Inclusion and Exclusion Criteria

Inclusion criteria for the analysis included (a) age of 18 or older; (b) the presence of supratentorial CSDH via computed tomography (CT); (c) Markwalder grading scale (MGS) and Glasgow Coma Scale (GCS) grade of 0–2 (Table 1); and (d) acceptance of a 24-week follow-up period. We excluded patients with (a) a high risk of cerebral hernia and/or the need for immediate surgical intervention; (b) allergy to atorvastatin or other statins; (c) deranged liver function or uncontrolled hepatitis; (d) statin treatment over the previous 6 months; (e) a diagnosis of cancer; (f) treatment with prophylactic antiplatelet medications; and (g) previous CSDH surgery. Mild CSDH was defined when brain CT scan reveals slight compression of the brain parenchyma caused by hematoma and patients have no neurological symptoms or have moderate symptoms with MGS-GCS 0–2.

**TABLE 1:** Patients were evaluated using the Glasgow Coma Scale and Markwalder’s Grading Scale.

**Patient’s grade**	**Glasgow Coma Scale**	**Markwalder’s Grading Scale**
Grade 0	Glasgow Coma Scale score of 15	Normal neurological status without any symptoms
Grade 1	Glasgow Coma Scale score of 15	Without neurological deficits, but with symptoms such as headache or unsteady gait
Grade 2	Glasgow Coma Scale score of 13 to 14	Focal neurological deficits, such as drowsiness or disorientation, or variable neurological deficits, such as hemiparesis
Grade 3	Glasgow Coma Scale score of 9 to 12	With stupor but appropriate responses to noxious stimuli and several focal neurological signs such as hemiplegia
Grade 4	Glasgow Coma Scale score of less than 9	Coma with absent motor responses to noxious stimuli and decerebrate or decorticate posturing

*Only patients with Grades 0–2 CSDH were selected for atorvastatin treatment in this study.*

### Atorvastatin Therapy

All included patients were scheduled to receive an oral dose of 20 mg atorvastatin daily (Pfizer, United States) for 8 weeks (Bakker-Arkema et al., 1996; Cilla et al., 1996), without other special medications which could affect hematoma resolution such as mannitol, steroids, tranexamic acid and angiotensin converting enzyme (ACE) inhibitors etc. This dosage was chosen because it has been reported to be the most potent dose to facilitate angiogenesis without the risk of hemorrhage (Wang et al., 2014). All patients and/or their family members were thoroughly informed of the risks and benefits of non-surgical treatment with ATO. Patients would be switched to surgery to remove the hematoma if any worsening of symptoms, deterioration in the GCS, or new focal neurological deficits occurred or if CT indicated an increase in the size of CSDH during the follow-up period. Patients were monotherapied by ATO due to mild/none symptoms and their denial for operation.

### Evaluation and Follow-Up

We defined poor therapeutic efficacy (no response) as the need to switch to surgery due to deterioration in the neurological state and/or expansion of the hematoma volume, and we defined good therapeutic efficacy (response) as improvement in the neurological state and/or reduced or stable hematoma volume during the 24-week follow up that did not necessitate a change in therapy.

Regular radiographic follow-up visits at 4, 8, 12, and 24 weeks were recommended. Complete blood cell counts and serum levels of alanine aminotransferase, aspartate transaminase, gamma glutamyl transpeptidase, urea nitrogen, and creatinine were measured at baseline and at the 4th, 8th, and 24th weeks to evaluate hematological, liver, and kidney function.

**FIGURE 1:**
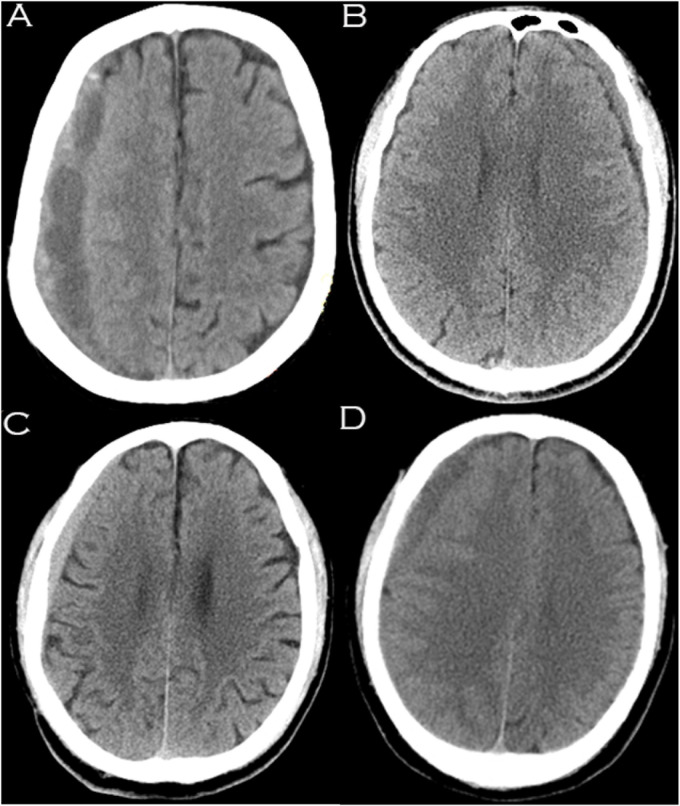
Hematomas of different densities as visualized on computed tomography images. **(A)** Mixed density hematoma; **(B)** Isodensity hematoma; **(C)** Hyperdensity hematoma; **(D)** Low-density hematoma.

Information regarding patients’ age, sex, weight, height, blood pressure, duration from symptoms to trauma, location of CSDH (unilateral or bilateral), cause of traumatic brain injury (TBI), headache scale, Markwalder grading scale, ADL-BI, American Society of Anesthesiologists Physical Status classification system (ASA-PS), distance of midline shift (mm), basal cistern compression, initial maximal thickness of the subdural hematoma (mm) and the presence of organized hematoma was recorded and assessed. Basal cistern compression was defined if the basal cisterns were obliterated and/or compressed on the first CT scan (Supplementary Figure 1). The midline shift was identified as deviation of the septum pellucidum from the central position (Eisenberg et al., 1990). The subdural hematoma density was classified into the 4 following groups based on CT findings: low density (<25 Hounsfield units [HU]), isodense (25–35 HU), hyperdense (>35 HU), and mixed density (Kim et al., 2017; Figure 1). A hematoma volume was calculated based on the Coniglobus Formula given as: hematoma volume (ml) = 1/2 × the longest diameter of the hematoma layer with the largest area (cm) × the longest diameter perpendicular to the longest diameter (cm) × the thickness of the hematoma (cm). If a patient had more than one hematoma, a total volume of multiple hematomas was calculated.

### Statistical Analyses

Univariate analysis was performed with the Pearson X^2^ test, continuity correction X^2^ test or Fisher’s exact test (sex, TBI history, cause of TBI, duration from symptoms to traumatic history, basal cistern compression, bilateral hematoma, density of the subdural hematomas, and organized hematoma). The Mann-Whitney *U* test was used for ordinal categorical variables (MGS-GCS, ADL-BI scoring ASA-PS and headache scale). The independent-samples Student’s *t* test was used to analyze non-categorical variables (age, weight, height, blood pressure, midline shift, thickness of hematoma, volume of hematoma, blood cell counts, and serum levels). Multivariate analysis with a logistic regression model was used to determine independent associations among the factors indicative of no response to ATO as a monotherapy for CSDH in the univariate analysis. The relationship between each predictive factor and the effect of ATO on CSDH is expressed as the odds ratio (OR) and 95% confidence interval (CI). A *p* value < 0.05 was considered statistically significant. Receiver operating characteristic (ROC) curves were produced, and 95% confidence intervals were determined. The ROC curve analysis generated areas under the curve (AUCs), which were used to estimate the predictive capacity of probable risk factors in our study. Statistical analysis was performed using SPSS statistics software for Mac (SPSS Version 25.0, IBM Corporation, Armonk, NY, United States).

## Results

Of the 95 patients, 89 patients with mild or moderate CSDH completed the 24-week follow-up (75 men and 14 women; age range, 24–88 years; mean age, 61.95 ± 15.30 years); 5 patients were lost to follow-up, and 1 patient died of pulmonary embolism. Among the patients who completed follow-up, 82 patients had experienced apparent head trauma; the median interval between trauma and the first symptom was 1–2 months. The main symptoms and signs of CSDH were headache, dizziness, weakness, dysphoria, irritability and gait disturbance. According to the MGS-GCS scale, 3, 80, and 6 patients were grade 0, 1, and 2, respectively, at admission. These patients were classified as independent (55 patients), slightly dependent (25 patients), moderately dependent (4 patients), severely dependent (3 patients) and completely dependent (2 patients) according to the baseline ADL-BI before the treatment (Table 2). Thirty two patients (36.0%) had a medical history of hypertension (*n* = 15, 16.9%), hyperlipidemia (*n* = 6, 6.7%), coronary heart disease (*n* = 2, 2.2%), hemorrhoids (*n* = 1, 1.1%), epilepsy (*n* = 1, 1.1%), chronic bronchitis (*n* = 1, 1.1%), lacunar infarction (*n* = 1, 1.1%), migraine (*n* = 1, 1.1%), diabetes mellitus (*n* = 1, 1.1%), hepatitis B (*n* = 1, 1.1%), atrial fibrillation (*n* = 1, 1.1%), and hernia (*n* = 1, 1.1%).

**TABLE 2:** Baseline characteristics of patients with CSDH enrolled in the study.

**Characteristic**	**Number of patients (%)**
Age, median	65.0(24.0−88.0)
Male	75 (84.3)
Body height, median, cm	170.0(145.0−180.0)
Weight, median, kg	80.0(56.0−110.0)
CSDH with TBI history	82 (92.1)
Impact of motor vehicles, bicycles, etc.	13 (14.6)
Fall	33 (37.0)
Bump	34 (38.2)
Sports / recreation	1 (1.1)
Raid	3 (3.4)
Drop	4 (3.4)
Other	5 (3.4)
Duration from trauma to symptoms	
3 weeks-1 month	32 (36.0)
1–2 months	31 (34.8)
2–3 months	13 (14.6)
3–6 months	4 (4.5)
>6 months	2 (2.2)
MGS-GCS score	
0	3 (3.4)
1	80 (89.9)
2	6 (6.7)
ADL-BI score	
95–100 independent	55 (61.8)
80–90 slightly dependent	25 (28.1)
65–75 moderately dependent	4 (4.5)
45–60 severely dependent	3 (3.4)
0–40 completely dependent	2 (2.2)
Headache	
none	26 (29.2)
Minor: can be endured, does not affect living	36 (40.4)
Moderate: can still be endured, affect living	26 (29.2)
Severe: affect living, patient must break even be in bed	1 (1.0)
Density of the subdural hematomas	
Isodensity hematoma	13 (14.6)
Low-density hematoma	28 (31.5)
High-density hematoma	26 (29.2)
Mixed density hematoma	22 (24.7)
Mean total hematoma volume, ml	65.9(8.7−135.1)
Mean midline shift, mm	3.4(0−15.0)
Mean maximal thickness of hematoma, mm	15.6(3.0−31.0)
Basal cistern compression	23 (258)
Organized hematoma	11 (12.4)
RBC, median, 10^12/L	4.59(2.79−5.49)
HGB, median, g/L	140(85−166)
WBC, median, 10^9/L	6.64(3.74−12.9)
PLT, median, 10^9/L	216(105−400)
APTT, median, s	31.6(19.3−45.3)
PT, median, s	11.8(9.1−14.3)
FIB, median, g/L	3.08(1.3−6.33)
D-Dimer, median, mg/L	0.48(0.01−18.7)
INR, median	0.98(0.79−1.14)
Lipemia, median, mmol/l	1.21(0.33−8.3)

*ADL-BI, Activities of Daily Life-the Barthel Index; CSDH, chronic subdural hematoma; MGS-GCS, Markwalder grading scale–Glasgow Coma Scale; TBI, traumatic brain injury; WBC, white blood cell; INR, international normalized ratio; FIB, fibrinogen; APTT, activated partial thromboplastin time; PT, prothrombin Time; RBC, red blood cell; HGB, hemoglobin.*

Computed tomography findings on admission identified 13 patients with an isodense hematoma (14.6%), 28 patients with low-density hematoma (31.5%), 26 patients with hyperdense hematoma (29.2%), and 22 patients with mixed-density hematoma (24.7%). The CSDH was bilateral in 6 patients. Basal cistern compression (BCC) was observed in 23 patients. The midline shift ranged from 0 to 15 mm (3.44 ± 3.90 mm). The maximum thickness of the hematoma (MTH) ranged from 3 to 31 mm (15.58 ± 5.17 mm), and 11 of 89 (12.4%) patients had organized hematoma. CSDHs were located in the frontal (*n* = 4), frontotemporal (*n* = 1), frontoparietal (*n* = 34), bilateral (*n* = 19) and lateral cerebral hemispheres (*n* = 31). The volume of hematoma (VOH) ranged from 8.7 to 135.1 ml (65.9 ± 31.23 mm) (Table 2).

Among the 89 patients, 11 (11/89, 12.36%) were switched to surgery during the follow-up period due to a deteriorating neurological state and enlarged hematoma on CT scan; of them, 8, 1, 1, and 1 patients presented with hyperdense, hypodense, isodense and mixed-density hematomas, respectively. These patients were regarded as having a poor therapeutic response and were switched to surgery over a range of 2–27 days with a median interval of 12 days after the first day of medication.

The remaining 78 patients who received ATO for 8 weeks and were followed up for another 16 weeks were regarded as having a good response to pharmacotherapy, including 9 who refused CT scans because they were asymptomatic with declining trend in hematoma volume. At last follow-up brain CT, SDH had nearly disappeared in 59 patients. Hematoma is visible in 11 patients (range, 1.5–27.5 ml; mean, 11.25 ± 6.76 ml) with MTH ranged from 0.7 to 9 mm (4.97 ± 2.42 mm). All 11 of patients, midline shift and BBC are invisible (Table 3[[AQ16]]). All patients who were switched to surgery underwent a burr hole craniostomy with drainage. The demographic information of the patients who were switched to surgery is listed in Table 4. During the course of ATO therapy, there were no significant changes in blood cell counts or hematological and liver function. No documented ATO-related side effects were observed.

**TABLE 3:** Information of patients with visible CSDH at the end of follow up.

**Age (years)**	**Sex**	**VOH ml**		**MTH mm**		**MLS mm**		**BCC**		**MGS-GCS**		**ADL-BI**		**Headache**	
		**Baseline**	**EOF**	**Baseline**	**EOF**	**Baseline**	**EOF**	**Baseline**	**EOF**	**Baseline**	**EOF**	**Baseline**	**EOF**	**Baseline**	**EOF**
57	male	65	1.5	18	0.7	0	0	no	no	1	1	95	100	2	1
74	male	101.7	7.3	16	4	0	0	no	no	1	0	80	100	4	4
61	male	82.3	9.5	21	5	5	0	no	no	1	0	100	100	1	4
75	male	101.8	8.8	25	5	6	0	yes	no	1	0	100	100	1	4
83	female	86.5	18.3	14	5	5	0	no	no	1	0	90	100	2	4
72	male	34.3	11.2	12	5	0	0	no	no	1	0	90	100	4	4
68	female	107.5	8.8	22	7	15	0	no	no	2	0	90	100	1	4
65	male	100.3	27.5	12	9	0	0	no	no	1	1	95	100	2	1
75	male	81.7	13.2	19	8	7	0	no	no	2	0	45	100	1	4
61	male	64	9.6	15	2	0	0	no	no	1	0	25	100	1	4
83	male	27.9	8	9	4	0	0	no	no	1	1	100	100	1	1

*BCC, basal cistern compression; MTH, maximum thickness of the hematoma; VOH, volume of hematoma; MLS, midline shift; EOF, end of follow-up; ADL-BI, activities of daily living–Barthel Index; MGS-GCS, Markwalder grading scale–Glasgow Coma Scale.*

**TABLE 4:** Demographic information of patients who were switched to surgery.

**Age (years)**	**Sex**	**Cause of trauma**	**Traumatic history (month)**	**MGS-GCS score**	**ADL-BI score**	**Headache**	**Hemorrhage Side**	**VOH (ml)**	**Midline Shift (mm)**	**BCC**	**Hematoma Density**	**MTH (mm)**
63	male	Fall	3 weeks-1 month	1	100	Minor	Right	90.9	7	Yes	Hyper	16
53	male	Bump	3 weeks-1 month	1	100	Moderate	Right	42.8	5	No	Hyper	13
72	male	Fall	1–2 months	1	100	Moderate	Left	98	9	Yes	Hyper	20
42	male	Impact of motor vehicles, bicycles, etc.	2–3 months	0	100	None	Left	52.2	0	No	Hypo	12
51	male	Fall	1–2 months	1	100	Minor	Left	109	9	Yes	Hyper	20
24	female	Sports/Recreation	1–2 months	1	100	Moderate	Bilateral	133	0	No	Homo	25
57	male	Bump	3 weeks–1 month	1	100	Moderate	Right	79	10	Yes	Hyper	19
72	female	Bump	2–3 months	1	100	Moderate	Left	117	12	Yes	Hyper	28
63	male	Raid	3 weeks–1 month	1	95	Moderate	Right	68.7	7	Yes	Hyper	17
68	male	None	None	1	95	None	Right	124.5	9	Yes	Hyper	21
65	male	Bump	1–2 months	1	95	Moderate	Left	115.5	14	Yes	Mixed	19

*BCC, basal cistern compression; MTH, maximum thickness of the hematoma; VOH, volume of hematoma.*

Patients were categorized into two groups based on whether they were respond to ATO treatment or not. Univariate analysis showed that the effect of ATO was significantly associated with BCC (*p* = 0.001), hyperdense hematoma (*p* = 0.03), the ADL-BI score (*p* = 0.007), midline shift (*p* = 0.001), VOH (*p* = 0.01) and MTH (*p* = 0.016). Multivariate logistic regression analysis identified hyperdense hematoma (OR, 0.474; 95% CI, 0.249–0.902; *p* = 0.023), VOH (OR, 1.041; 95% CI, 1.007–1.076; *p* = 0.018) and basal cistern compression (OR, 9.685; 95% CI, 1.761–53.257; *p* = 0.009) as independent risk factors for the outcome of atorvastatin treatment for CSDH. The results of univariate and multivariate analyses for the identification of predictive factors for the efficacy of atorvastatin monotherapy for CSDH are shown in Table 5.

**TABLE 5:** Factors related to outcome of atorvastatin as a monotherapy for chronic subdural hematoma: univariate analysis and multivariable analysis.

**Factor**	**Number of patients (%)**		***p* value, univariable analysis**	***p* value, multivariable analysis**	**OR (95% CI), multivariable analysis**
	**Respose**	**No response**			
Total	78	11			
Sex			1.000	0.571	0.523 (0.55–4.931)
Male	66 (83.9%)	9 (84.6%)			
Female	12 (16.1%)	2 (18.1%)			
Age (years)	62.55 ± 15.43	57.27 ± 14.40	0.288	0.082	0.954 (0.905–1.006)
Systolic pressure, mmHg	130.76 ± 12.82	131.73 ± 9.0	0.809	0.955	1.002 (0.928–1.082)
Diastolic pressure, mmHg	80.06 ± 9.82	80.45 ± 8.99	0.901	0.929	0.995 (0.898–1.103)
Body height (cm)	166.23 ± 20.15	167.27 ± 4.50	0.865	0.601	1.076 (0.819–1.413)
Weight (kg)	66.51 ± 12.11	66.82 ± 9.27	0.937	0.719	1.026 (0.897–1.173)
Traumatic brain injury history	72 (92.3%)	10 (90.9%)	0.36	0.419	0.309 (0.018–5.326)
Different cause of TBI			0.41	0.658	1.017 (0.658–1.572)
Impact of motor vehicles, bicycles, etc.	12 (15.4%)	1 (9.1%)			
Fall	26 (33.3%)	3 (27.3%)			
Bump	26 (33.4%)	4 (36.4%)			
Sports / recreation	0	1 (9.1%)			
Raid	2 (2.6%)	1 (9.1%)			
Drop	3 (3.9%)	0			
Other	3 (3.9%)	0			
Headache	54 (69.2%)	9 (81.8%)	0.582	0.560	1.261 (0.577–2.758)
None	24 (30.8%)	2 (18.2%)			
Minor: can be endured, does not affect living	34 (43.6%)	2 (18.2%)			
Moderate: can still be endured, affect living	19 (24.4%)	7 (63.6%)			
Severe:affect living, patient must break even be in bed	1 (1.3%)	0			
RBC, 10^12/L	4.53 ± 0.51	4.76 ± 0.44	0.157	0.597	NA
HGB, g/L	139.59 ± 14.51	142.27 ± 19.57	0.591	0.413	1.092(0.885–1.346)
WBC, 10^9/L	7.03 ± 1.84	7.36 ± 3.13	0.747	0.848	0.948(0.550–1.635)
PLT, 10^9/L	225.82 ± 65.08	196.55 ± 53.48	0.161	0.867	1.002(0.984–1.020)
APTT, s	31.88 ± 5.55	29.42 ± 5.62	0.177	0.063	0.707(0.490–1.020)
PT, s	11.82 ± 1.18	12.05 ± 1.14	0.555	0.324	1.343(0.748–2.414)
FIB, g/L	3.30 ± 0.94	2.90 ± 1.49	0.23	0.146	0.584(0.282–1.207)
D-Dimer, mg/L	0.86 ± 1.64	3.70 ± 7.38	0.39	0.393	1.197(0.793–1.806)
INR	0.98 ± 0.07	0.97 ± 0.08	0.719	0.174	NA
Lipemia, mmol/l	1.59 ± 1.37	1.57 ± 1.32	0.967	0.504	0.824(0.466–1.455)
ASA-PS			0.719	0.883	0.901(0.225–3.604)
1	47 (60.3%)	6 (54.5%)			
2	31 (39.7%)	5 (45.5%)			
Duration from trauma to symptoms			0.710	0.255	0.642(0.299–1.377)
3 weeks-1 month	28 (35.9%)	4 (36.4%)			
1–2 months	27 (34.6%)	4 (36.5%)			
2–3 months	11 (14.1%)	2 (18.2%)			
3–6 months	4 (51.3%)	0			
>6 months	2 (2.6%)	0			
MGS-GCS scale			0.167	0.124	0.119(0.008–1.794)
0	2 (2.6%)	1 (9.1%)			
1	70 (89.7%)	10(90.9%)			
2	6 (7.7%)	0 (0%)			
ADL-BI scoring	89.71 ± 15.16	98.64 ± 2.33	0.007*	0.996	1.232 (0.949–1.601)
Midline shift (mm)	2.87 ± 3.58	7.45 ± 4.41	0.001*	0.53	1.100 (0.820–1.476)
Basal cistern compression	15 (19.2%)	8 (72.7%)	0.001*	0.009*	9.685 (1.761–53.257)
Bilateral hematoma	18 (23.1%)	1 (9.1%)	0.558	0.696	0.623(0.058–6.666)
Isodensity hematoma	12 (15.4%)	1 (9.1%)	1.000	0.263	0.394(0.077–2.012)
Low-density hematoma	27 (34.6%)	1 (9.1%)	0.12	0.559	0.735(0.261–2.068)
High-density hematoma	18 (23.1%)	8 (72.7%)	0.03*	0.023*	0.474 (0.249–0.902)
Mixed density hematoma	21 (26.9%)	1 (9.1%)	0.397	2.585	2.585(0.287–23.288)
Organized hematoma	11 (14.1%)	0 (0%)	0.4	0.999	NA
Volume of hematoma (ml)	61.98 ± 29.54	93.69 ± 29.88	0.001*	0.018*	1.041 (1.007–1.076)
Maximal thickness of hematoma (mm)	14.90 ± 4.95	19.09 ± 4.70	0.01*	0.43	1.108 (0.859–1.43)

OR, odds ratio; CI, confidence interval; NA, not available; ADL-BI, Activities of Daily Life-the Barthel Index; CSDH, chronic subdural hematoma; MGS-GCS, Markwalder grading scale–Glasgow Coma Scale; WBC, white blood cell; INR, international normalized ratio; FIB, fibrinogen; APTT, activated partial thromboplastin time; PT, prothrombin Time; RBC, red blood cell; HGB, hemoglobin. ASA-PS, American Society of Anesthesiologists Physical Status classification system; TBI, traumatic brain injury. *Significant difference.

**FIGURE 2:**
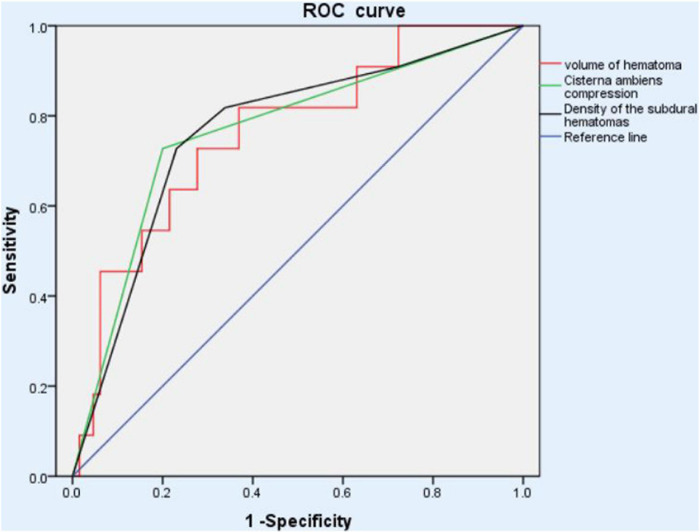
ROC analysis of different outcome to atorvastatin monotherapy showed that the basal cistern compression, volume of hematoma and hyper-density had the AUCs, 0.764 (*p* = 0.005), 0.762 (*p* = 0.006), and 0.748 (*p* = 0.009), respectively, suggesting medium predictability for therapeutic effect. ROC, receiver operating characteristic.

Areas under the curve were calculated for the good therapeutic efficacy and poor therapeutic efficacy groups after drug treatment, and BCC, VOH and hyperdensity were found to have AUC values of 0.764 (*p* = 0.005), 0.762 (*p* = 0.006), and 0.748 (*p* = 0.009), respectively; these AUCs indicate low (<0.70), medium (0.70–0.90), and high (>0.90) accuracy for predicting the effect of atorvastatin therapy, respectively (Figure 2).

## Discussion

Treatment for CSDH in advanced-age patients who have mild symptoms, take long-term anticoagulation/antiplatelet medications, or have poor physical health carries a considerable risk of complications, which could become a problem. From the literature, it is apparent that conservative therapy for CSDH could be used in some cases reducing the number of unnecessary operations due to operative or anesthetic complications (Santarius and Hutchinson, 2004; Xu et al., 2016; Soleman et al., 2017; Jiang, 2018). Effective and low-cost non-surgical atorvastatin treatment was found to significantly improve the outcomes of patients with moderate CSDH in our prior study (Jiang, 2018). However, to date, no studies in the literature have analyzed the prognostic factors of atorvastatin monotherapy in subgroup CSDH. Atorvastatin was either performed alone, along with additional burr hole drainage, with other drugs or along with a craniotomy in varying severity CSDH cohort (Xu et al., 2016; Chan et al., 2017; Qiu et al., 2017; Tang et al., 2018). In this study, although many risk factors were included in our logistic regression model, we found that hyperdense hematoma, BBC, and VOH were independent predictors of the efficacy of atorvastatin for the non-surgical treatment of CSDH.

Chronic subdural hematoma is the result of a series of complex mechanisms thought to be initiated by a separation of the border cell layer in the dura, which triggers healing responses including cell proliferation in the dural border, granulation tissue formation, and macrophage deposition (Santarius et al., 2010). Accumulation of blood within the subdural space incites an inflammatory response, comprising fibroblast proliferation, granulation tissue formation, and release of angiogenic factors, which results in formation of a neomembrane (Frati et al., 2004; Santarius et al., 2010; Stanisic et al., 2012). Weir and Gordon (1983) found high concentrations of profibrinolytic substances, including fibrin degradation products and antiplasmin, which are important in maintaining the vicious cycle of bleeding, coagulation and rebleeding (Weigel et al., 2001). Hematoma stability is said to vary depending on the stage of this bleeding cycle (Yamashima et al., 1983). Therefore, we suspect that the different hematoma densities may be influenced by the cycle mentioned above, which has different levels of inflammatory mediators (Gandhoke et al., 2013). In clinical cases, CT remains the preferred radiological tool for CSDH diagnosis. CT-confirmed CSDH may present a variety of imaging characteristics, and it is well known that the radiological subtype of CSDH may change over time (Cilla et al., 1996). The radiological subtypes that can present at the time of diagnosis possibly represent different pathophysiological stages of CSDH (Jensen et al., 2020). It is thought that SDHs follow general evolution overtime, starting as hyperdense on CT, becoming isodense and finally becoming hypodense (Bergström et al., 1977; Scotti et al., 1977; Reed et al., 1986). If high-density hematomas represent an early step in CSDH development where bleeding is ongoing, this would explain the significantly higher rate of switching to surgery in the high-density hematoma group compared with the other three hematoma density groups. Additionally, the meningeal lymphatic vessel (mLV) drainage pathway is a newly discovered system that takes part in the clearance of waste products in the central nervous system (Alitalo, 2011; Wiig and Swartz, 2012; Aspelund et al., 2016). Subdural hematomas (SDHs) are characterized by rapidly or gradually accumulating hematomas between the arachnoid and dura mater. Liu et al. (2020) proved that SDH was absorbed through the mLV drainage pathway. Compared to the lower types of hematoma density, there are reasons to suspect that certain components of hyperdense hematomas could inhibit the function of mLVs (Yu et al., 2019).

The preoperative hematoma thickness and midline shift have been reported as prognostic factors of CSDH, consistent with our results in the univariate analysis (Torihashi et al., 2008; Stanišić et al., 2013). However, our results only found a significant relationship between hematoma volume and the outcome of atorvastatin monotherapy for CSDH in the multivariate analysis. We hypothesize that the degree of compression from hematoma may be a result of multiple factors, including hematoma thickness, midline shift, patient age, and brain atrophy (Fujitani et al., 2017). In our present study, under the premise of brain atrophy in the elderly, hematoma volume and basal cistern compression may contribute more directly to intracranial pressure growth and neurological dysfunction.

In our univariate analysis, the ADL-BI score, which measures the daily living activities of patients with neurological diseases, was more sensitive than the Markwalder grading scale/Glasgow Coma Scale and ASA-PS for atorvastatin treatment prognosis. The American Society of Anesthesiologists Physical Status classification system is a method of characterizing patient operative risk on a scale of 1–5, with 1 being normal health and 5 being moribund, that has strong, independent associations with postoperative medical complications and mortality (Hackett et al., 2015). The GOS was originally developed to evaluate the recovery of patients with traumatic brain injury, while the ADL-BI scores, measuring the daily living ability to go up and down a flight of stairs, may indirectly reveal more remove information regarding the outcomes of our non-surgical cohort.

Receiver operating characteristic (ROC) analysis is a tool used to describe the discrimination accuracy of prediction models (Obuchowski and Bullen, 2018). With the use of this sophisticated statistical analysis, we estimated the predictive capacity of the known independent risk factors in our study. The AUCs of our three independent risk factors were all under 0.90, which implies that the relationship between these risk factors and drug treatment efficiency is more of an association than a causal relation. There may be more complex interacting factors at play that are out of our view. In our center, we relied on clinical manifestations of patients (MGS-GCS 0–2) rather than just the hematoma volume. Many patients are old and have significant brain atrophy, and sometimes a relatively large hematoma does not necessarily mean that the patient is in poor condition and needs immediate surgery. In younger, long-term oral aspirin and warfarin patients, as they are more likely to develop an acute condition, doctors should consider their management carefully. When evaluation difficulties are encountered, such as patients with relatively large hematoma or midline shift, we require early hospitalization and close monitoring of patients. If the rating of the scale still increases and/or the condition deteriorates after taking the conservative treatment, we will carry out surgical treatment in time.

By reason of communicating small vessels penetrate from the middle meningeal artery (MMA) through the dura mater and connect to the newly developed neo-vessels in the outer membrane of the CSDH (Tanaka and Kaimori, 1999). Rupture of these neo-vessels may further enlarge the hematoma (Takizawa et al., 2016). Hence, eliminating the blood supply to the outer neomembrane by embolization of the MMA (eMMA) has been proposed as a minimally invasive treatment for CSDH (Ban et al., 2018; Catapano et al., 2020, 2021a, 2021b). And several systematic reviews and a meta-analysis have shown promising results with MMA embolization, including low rates of complication and recurrence (Haldrup et al., 2020; Ironside et al., 2021). However, variation of treatment regimens of eMMA is problematic. Embolization was conducted either in isolation, in combination with surgical intervention or with drugs. Also, different embolization agents, extent of penetration, patient selection, timing of embolization and number of MMA branches embolized were used in the different studies (Catapano et al., 2020, 2021a). Moreover, symptoms and neurological deficits from CSDH arise most often due to the mass effect of the subdural collection. Whereas, similar to atorvastatin treatment, eMMA is not able to reduce intracranial pressure and thereby alleviate symptoms and deficits in the acute phase, which is an obvious drawback of all conservative treatments for enlarging or symptomatic CSDH compared with surgery. So we suggest that future studies of conservative management for CSDH should focus more on subgroup analysis, like this mild CSDH subgroup study, which is prone to similar baseline characteristics and may elucidate more information about appropriate patient selection.

Our study has a retrospective design, which usually produces a risk of bias. In the multivariate model, although ideally, more stringent criteria for variable inclusion in the model would be applied in lieu of *p* < 0.1, this may lead to too few variables and too many confounding factors, which will have a greater impact on the results. An association between a specific radiological subtype and a specific event of CSDH may be difficult to determine, as a large part of CSDH pathogenesis remains unclear – even whether a head injury is required for the formation of a CSDH is still an open question (Jensen et al., 2020). Despite these limitations, this study still provides useful information to predict the effects of atorvastatin on CSDH patients receiving non-surgical treatment, which can provide a drug treatment strategy for neurosurgeons and reduce the requirement for surgical intervention in appropriately selected patients.

## Conclusion

Atorvastatin monotherapy is effective for the treatment of mild or moderate CSDH. High-density hematoma, BBC, and hematoma volume are independent predictors of a deteriorating neurological state and/or expansion of the hematoma volume in patients receiving atorvastatin as a non-surgical treatment. The ADL-BI might be more sensitive than the MGS-GCS scale and ASA-PS for determining the outcomes of mild or moderate CSDH patients. These findings allow for individual risk assessments and might prompt clinicians to tailor treatment measures.

## Data Availability Statement

The raw data supporting the conclusions of this article are available from the corresponding authors by request.

## Ethics Statement

Written informed consent was obtained from the individual(s) for the publication of any potentially identifiable images or data included in this article.

## Author Contributions

RJ and JZ designed this study and revised the final version of the manuscript. XZ, DW, and YT carried out this study, collected, and analyzed the data. HW, XL, TX, YF, and CG helped in data collection. JH, ZS, and WQ helped to analyze the results. XZ wrote the draft. All authors contributed to the article and approved the submitted version.

## Conflict of Interest

The authors declare that the research was conducted in the absence of any commercial or financial relationships that could be construed as a potential conflict of interest.

## Publisher’s Note

All claims expressed in this article are solely those of the authors and do not necessarily represent those of their affiliated organizations, or those of the publisher, the editors and the reviewers. Any product that may be evaluated in this article, or claim that may be made by its manufacturer, is not guaranteed or endorsed by the publisher.
