# Supplementary material for: Risk Factors for Atorvastatin as a Monotherapy for Chronic Subdural Hematoma: A Retrospective Multifactor Analysis
Source: Front Aging Neurosci. 2021 Sep 1;13:726592. doi: 10.3389/fnagi.2021.726592 (PMC8440973; doi:10.3389/fnagi.2021.726592)
Supplement: Supplementary file 1 [file Image_1.pdf]

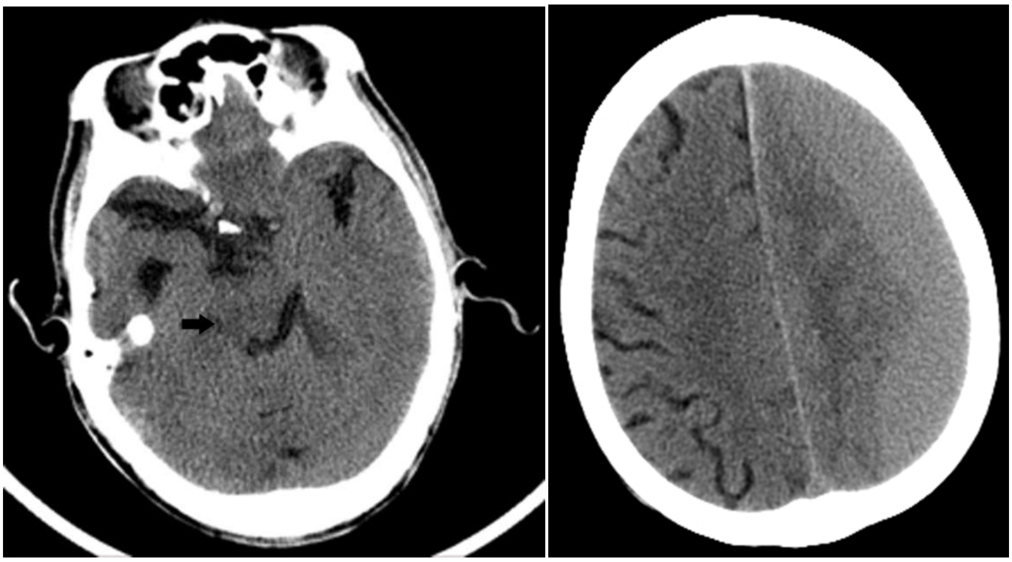

Supplementary figure: Two CT scans of the same patient. The black arrow indicates the basal cisterns were obliterated and/or compressed.
